# Hyaluronic Acid and β-Tricalcium Phosphate in Periodontal Pocket Therapy and Alveolar Bone Augmentation: A Systematic Review

**DOI:** 10.3390/dj14020097

**Published:** 2026-02-10

**Authors:** Andrea Bors, Liana Beresescu, Felicia Gabriela Beresescu

**Affiliations:** Faculty of Dentistry, George Emil Palade University of Medicine, Pharmacy, Science, and Technology, 38 Gheorghe Marinescu Street, 540142 Târgu Mureș, Romania; liana.beresescu@umfst.ro (L.B.); felicia.beresescu@umfst.ro (F.G.B.)

**Keywords:** alveolar augmentation, biomaterials, bone regeneration, β-tricalcium phosphate, hyaluronic acid, periodontal regeneration, ridge preservation, soft-tissue healing

## Abstract

**Background:** Hyaluronic acid (HA) and β-tricalcium phosphate (β-TCP) are widely used biomaterials in periodontal and alveolar regeneration; however, their complementary biological roles across soft- and hard-tissue healing have not been jointly assessed in a single review. **Objective:** to systematically evaluate clinical and translational evidence regarding the adjunctive use of HA in periodontal therapy and the regenerative performance of β-TCP in alveolar bone reconstruction. **Methods:** A systematic search was conducted across PubMed/MEDLINE, Scopus, Web of Science Core Collection, and Embase for studies published between 1 January 2015 and 1 October 2025. Randomized and non-randomized clinical studies evaluating HA as an adjunct to periodontal therapy and β-TCP in ridge preservation or augmentation were included. In vitro studies were considered when providing mechanistic insight relevant to clinical outcomes. Screening, data extraction, and qualitative synthesis were performed according to PRISMA 2020 guidelines. **Results:** Database searching identified 312 records. After removal of duplicates, 241 records were screened, of which 179 were excluded. Sixty-two full-text articles were assessed for eligibility, and twenty studies met the inclusion criteria (twelve clinical; eight in vitro). Across non-surgical periodontal therapy trials, adjunctive HA demonstrated modest but consistent additional improvements in probing depth reduction (~0.8–1.5 mm) and clinical attachment gain (~0.5–1.2 mm) compared with mechanical therapy alone, particularly in deeper defects and systemically compromised patients. Clinical studies on β-TCP reported predictable dimensional bone preservation and stable implant feasibility, supported by histologic evidence of scaffold-guided new bone formation. In vitro findings indicated that HA modulates biofilm-induced inflammation and supports fibroblast and epithelial cell function, whereas β-TCP promotes osteoblast activity and controlled osteoclast-mediated remodeling. **Conclusions:** HA and β-TCP demonstrate complementary regenerative roles, with HA primarily enhancing soft-tissue resolution and inflammatory modulation and β-TCP providing osteoconductive structural support for bone regeneration. Current evidence supports their selective integration in personalized regenerative approaches; however, standardized outcome reporting and longer-term trials are required to establish the clinical value of sequential or combined application.

## 1. Introduction

Periodontal pocket therapy and alveolar bone augmentation are critical in managing advanced periodontitis and preparing sites for implant placement. Periodontal regenerative therapies aim to reduce pocket depth and restore attachment, whereas clinical studies have shown that β-TCP supports predictable new bone formation and dimensional ridge preservation compatible with future implant placement [1,2]. In recent years, biologically active adjuncts such as hyaluronic acid (HA) and bone substitute biomaterials like β-tricalcium phosphate (β-TCP) have gained attention for their potential to enhance healing outcomes in these contexts [1,3,4,5].

Hyaluronic acid is a naturally occurring glycosaminoglycan abundant in the extracellular matrix of gingiva, periodontal ligament, and alveolar bone [2]. It is well known for its biocompatibility, biodegradability, and multifunctional role in wound healing [6,7]. Preclinical and clinical observations indicate that adjunctive application of HA in periodontitis can reduce inflammation and promote tissue repair [8,9,10]. Specifically, improvements in bleeding on probing, probing pocket depth (PPD) reduction, clinical attachment level (CAL) gain, and defect fill have been reported when HA is used alongside standard periodontal therapy [11,12,13]. Despite numerous studies, consensus on the magnitude of HA’s benefits in periodontal regeneration has been uncertain [14,15,16], partly due to variability in formulations (molecular weight and cross-linking) and application methods (irrigation, gels, and injections). A narrative review by Casale et al. noted enhanced tissue repair and patient quality of life with HA, though it emphasized the need for well-designed trials to confirm these benefits [16].

β-Tricalcium phosphate is a synthetic alloplastic bone-graft material composed of calcium and phosphate in a ratio similar to bone mineral [17]. β-TCP is purely osteoconductive—it provides a scaffold for new bone ingrowth—and is designed to resorb over time as natural bone replaces it. It has a long history of use in orthopedics and dentistry as an alternative or adjunct to autogenous bone grafts, avoiding donor site morbidity [17]. Numerous studies (animal and human) have demonstrated the bone regenerative efficacy of β-TCP, both when used alone and in composite grafts [18,19]. For example, in maxillary sinus floor augmentation—a common alveolar bone augmentation procedure—β-TCP alone has achieved new bone formation and implant survival rates comparable to those using autografts or other graft materials [1]. Long-term follow-up data show high success: one 10-year study reported ~97% implant survival after sinus augmentation with β-TCP, with an average ~7 mm vertical bone gain [20]. However, clinical outcomes using β-TCP have varied, and questions remain about its optimal use (e.g., need for combination with growth factors or membranes) and how it compares to gold-standard grafts in different defect types [21,22]. A recent network meta-analysis suggested that purely osteoconductive grafts like β-TCP can perform similarly to osteogenic or osteoinductive grafts in sinus augmentation [21,22,23,24,25,26], though results across studies are not uniform.

Given the growing interest in HA and β-TCP, a comprehensive evaluation of the evidence is needed. Previous reviews have addressed HA in periodontal therapy or β-TCP in bone grafting separately, but none have jointly considered their roles across periodontal and implant regenerative interventions. Moreover, integrating in vitro research can help elucidate the underlying mechanisms by which these materials exert their effects (e.g., cellular proliferation, differentiation, and antimicrobial action), informing clinical application. Therefore, the aim of this systematic review was to critically appraise and synthesize the current evidence (2015–2025) on (1) the efficacy of HA as an adjunct in periodontal pocket therapy; (2) the efficacy of β-TCP in alveolar bone augmentation for implant dentistry; and (3) relevant studies shedding light on their biological effects. We also evaluate the risk of bias and quality of the included studies and discuss the consistency of outcomes and remaining knowledge gaps.

## 2. Methods

A review protocol was specified a priori, outlining the eligibility criteria, search strategy, study selection procedures, data extraction methods, and approaches for quality assessment. Study screening, data extraction, and qualitative synthesis were conducted in accordance with the PRISMA 2020 (Preferred Reporting Items for Systematic Reviews and Meta-Analyses) guidelines. The completed PRISMA 2020 checklist is provided in the Supplementary Materials (File S1).

### 2.1. Protocol Registration

The review protocol was registered in the International Prospective Register of Systematic Reviews (PROSPERO) under the registration number *CRD420251268606*.

### 2.2. Information Sources and Search Strategy

Four electronic databases (PubMed/MEDLINE, Scopus, Web of Science Core Collection, and Embase) were systematically searched for studies published between 1 January 2015 and 1 October 2025. The search strategy combined controlled vocabulary (e.g., MeSH and Emtree) with free-text terms using Boolean operators. Search terms targeted the following two main concepts:

(1) Hyaluronic acid (HA) in periodontal therapy;
(2) β-Tricalcium phosphate (β-TCP) in alveolar bone augmentation.

For example, PubMed queries included terms for hyaluronic acid (e.g., “Hyaluronic Acid” OR “hyaluronan”) combined with periodontal terms (e.g., “periodont*” OR “periodontal pocket”) and β-TCP terms (e.g., “β-tricalcium phosphate” OR “β-TCP”) combined with bone augmentation terms (e.g., “alveolar ridge augmentation” OR “socket preservation”). Where appropriate, filters were applied for language and publication period. No restrictions were placed on publication type at the search stage. Reference lists of included studies and recent reviews were manually searched to identify additional eligible publications. The full electronic search strategies for all databases are provided in the Supplementary Materials (File S2).

### 2.3. Eligibility Criteria

Eligibility criteria were defined according to the PICOS framework for clinical studies and a simplified PI(O) approach for in vitro studies.

- Population (clinical studies):
  Patients diagnosed with chronic periodontitis or presenting periodontal intrabony defects (for HA studies) and patients requiring alveolar bone augmentation—such as ridge preservation or horizontal/vertical ridge augmentation prior to implant placement (for β-TCP studies). No restrictions were applied regarding age or sex.
- Interventions:
  Periodontal therapy: adjunctive application of HA in any formulation (e.g., gel and injection) used in non-surgical or surgical periodontal treatment.
  Bone augmentation: β-TCP used either alone or in combination with other materials for augmentation of extraction sockets or deficient ridges. Studies evaluating combined HA + β-TCP use were also eligible.
- Comparators:
  Standard periodontal therapy without HA or bone augmentation with alternative or no graft material. For single-arm studies reporting pre–post outcomes, a comparator was not required if quantitative outcome measures were presented. In vitro studies required appropriate control conditions.
- Outcomes:
  Periodontal studies: probing pocket depth (PPD), clinical attachment level (CAL), radiographic bone fill, or patient-reported outcomes where available.
  Bone augmentation studies: dimensional bone gain, percentage of vital bone, histological findings, and implant success or survival.
  In vitro studies: outcomes related to cell viability or proliferation, osteogenic or inflammatory marker expression, mineralized matrix formation, or antimicrobial effects.
- Study Types:
  Eligible designs included randomized or non-randomized controlled trials, controlled before–after studies, prospective or retrospective cohorts, case–control studies, and case series with ≥5 cases. In vitro studies were eligible if they evaluated HA or β-TCP in periodontal or bone-related contexts. Reviews, meta-analyses, animal studies, editorials, and single case reports were excluded, although their references were screened for primary sources. Only studies published in English were included.

### 2.4. Study Selection Process

**Figure 1:**
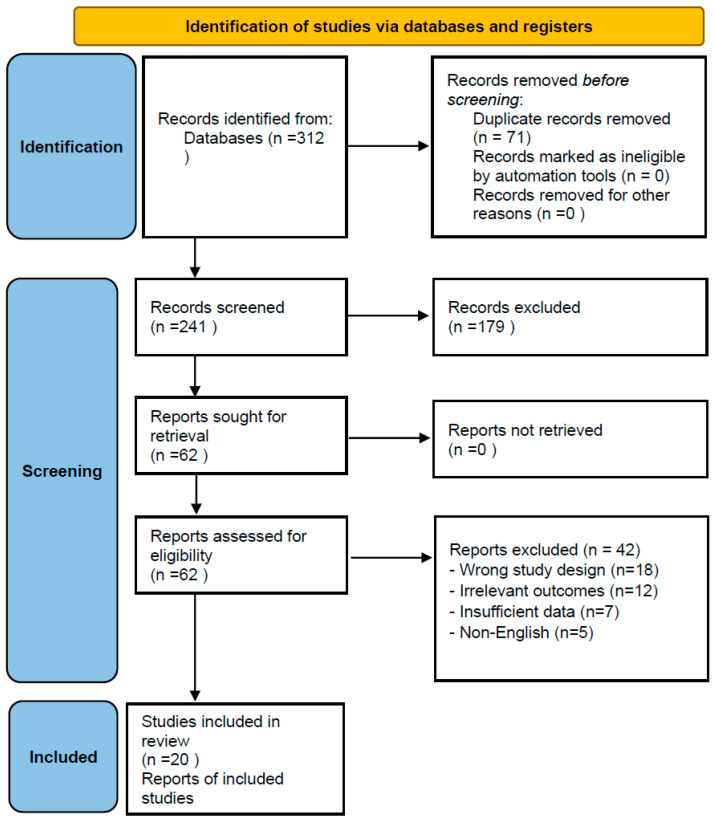
PRISMA flow diagram.

Two reviewers independently screened all retrieved records by titles and abstracts following a calibration phase to ensure consistency. Inter-reviewer agreement during the title and abstract screening phase was assessed using Cohen’s kappa statistic (κ = 0.83), indicating substantial agreement among reviewers, based on binary include/exclude decisions across 241 records. A complete list of excluded full-text articles with reasons for exclusion is provided in the Supplementary Materials (File S3). Reasons for exclusion were recorded. Disagreements were resolved through discussion and, if necessary, consultation with a third reviewer. The study selection process is illustrated in the PRISMA flow diagram (Figure 1). Certainty of evidence was not formally graded (e.g., GRADE), which is acknowledged as a limitation of this review.

### 2.5. Data Extraction

Data were extracted using a standardized form. For clinical studies, extracted information included study characteristics (author, year, country, and design), participant demographics, intervention and control details, follow-up duration, and quantitative outcomes. For in vitro studies, data included cell or tissue type, experimental model, concentrations or types of HA/β-TCP, measured outcomes, and primary findings. Data extraction was performed by one reviewer and cross-checked by a second reviewer. Authors were contacted for clarification when required, although no additional data were obtained. The study selection process is illustrated in the PRISMA flow diagram (Figure 1). A graphical version of the PRISMA flow diagram is also available in the Supplementary Materials (File S4).

### 2.6. Outcomes and Data Synthesis

Given the anticipated heterogeneity among interventions, study populations, and outcome measures, a narrative synthesis approach was adopted. Clinical studies were grouped into two categories—periodontal therapy with HA and alveolar bone augmentation with β-TCP—and outcomes were summarized descriptively. In vitro results were synthesized to contextualize potential biological mechanisms underlying clinical observations. Because of methodological diversity, no quantitative meta-analysis was performed and no statistical measures of heterogeneity were calculated. Accordingly, formal statistical assessment of heterogeneity (e.g., I^2^ statistics) and publication bias (e.g., funnel plot analysis) was not performed.

### 2.7. Risk of Bias and Quality Assessment

**Figure 2:**
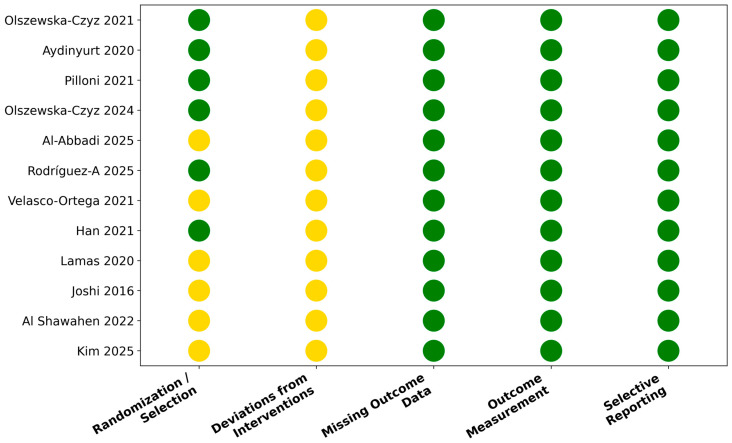
Risk of bias summary for included clinical studies. Randomized controlled trials were assessed using the Cochrane Risk of Bias 2.0 tool, while non-randomized studies were evaluated using the Newcastle–Ottawa Scale. Traffic-light colors indicate low risk (green) or some concerns (yellow). The studies included were: Olszewska-Czyz et al. (2021) [24], Aydinyurt et al. (2020) [25], Pilloni et al. (2021) [7], Olszewska-Czyz et al. (2024) [27], Al-Abbadi et al. (2025) [28], Rodríguez-A et al. (2025) [29], Velasco-Ortega et al. (2021) [30], Han et al. (2021) [31], Lamas et al. (2020) [32], Joshi et al. (2016) [33], Al Shawaheen et al. (2022) [34], and Kim et al. (2025) [12].

Risk of bias was independently assessed by two reviewers. Randomized controlled trials were evaluated using the Cochrane Risk of Bias 2.0 tool, while non-randomized or observational studies were assessed using the Newcastle–Ottawa Scale. In vitro studies were appraised based on methodological transparency, inclusion of appropriate controls, replicability of results, and relevance of the experimental model. A visual summary of risk of bias across clinical studies is provided in the main manuscript as a traffic-light plot (Figure 2). For Newcastle–Ottawa Scale assessments, studies were categorized as low risk or some concerns based on overall NOS performance across selection, comparability, and outcome domains. Detailed risk-of-bias and quality assessment data are provided in the Supplementary Materials (File S5).

## 3. Results

A total of 20 studies (12 clinical and 8 in vitro) were included in this systematic review, as outlined above. Table 1 and Table 2 summarize the key characteristics of the included clinical studies, and Table 3 presents an overview of the included in vitro studies. The clinical studies encompass a mix of randomized trials and controlled studies evaluating HA in periodontal therapy or β-TCP in bone augmentation, whereas the in vitro studies explore the cellular and microbiological effects of these materials. Below, we present the findings in the following two main sections: (A) clinical outcomes with HA (periodontal therapy) and β-TCP (alveolar bone augmentation), and (B) insights from in vitro studies. Risk of bias findings are noted where relevant and further discussed later.

Risk of bias across the included clinical studies is summarized in Figure 2. Most randomized trials showed low risk for outcome reporting but commonly presented unclear allocation concealment and limited blinding, which is difficult to achieve in local adjunctive periodontal interventions. Non-randomized studies showed variable methodological quality, with the most frequent limitations related to comparability of cohorts and potential confounding factors. In vitro studies demonstrated acceptable methodological consistency, although lack of standardized outcome measures limits cross-comparability.

## 4. Discussion

Periodontal regeneration and alveolar bone preservation remain central objectives in contemporary dental practice, as they directly influence long-term tooth retention, implant feasibility, and oral function. A wide range of materials and techniques have been proposed over the past decades, aiming to overcome the intrinsic biological limitations of healing after chronic periodontal inflammation, tooth extraction and bone loss. Clinical evidence has shown that periodontal regeneration is particularly challenging due to the combined need for soft-tissue resolution, bone reconstruction, and infection control [1,2,3]. The complexity of these processes explains why therapeutic adjuncts such as hyaluronic acid (HA) and beta-tricalcium phosphate (β-TCP) continue to receive interest, as they may assist biological processes that conventional mechanical or surgical treatment cannot fully address [4,5,6].

An important aspect when interpreting regenerative materials is the historical evolution of periodontal wound healing knowledge. Early models described periodontal repair as primarily a fibroblast-dominated process influenced by local contamination [7,8,9]. However, subsequent concepts emphasized cellular crosstalk between epithelial cells, periodontal ligament fibroblasts, alveolar osteoblasts, and the immune system [10,11,12]. These developments provided a biological rationale for adjunctive materials such as HA, known to regulate fibroblast migration, extracellular matrix hydration and inflammation [13,14,15,16]. Likewise, β-TCP became relevant as research clarified the role of osteoconduction and scaffold degradation in enabling structural bone replacement [17,18,19]. Thus, the foundation for combining soft-tissue modulators and osteoconductive scaffolds was laid conceptually long before current clinical protocols were established [20,21,22]. Importantly, evidence from orthopedic and craniofacial surgery demonstrates similar remodeling profiles of β-TCP in load-bearing contexts, thereby validating its translational relevance and not restricting its use to dental settings alone [40,41,42]. Clinical systematic reviews in implant dentistry further support the promising performance of β-TCP in sinus augmentation, ridge preservation, and simultaneous implant placement, with comparable implant survival rates and bone regeneration outcomes compared with other graft materials [21].

Clinically, mechanical debridement continues to represent the primary step in controlling chronic periodontitis. Nevertheless, adjunctive HA has been repeatedly shown to improve clinical outcomes beyond scaling and root planing alone [23,24,25,26]. Across trials, semi-quantified improvements of ~0.5–1.2 mm CAL gain and ~0.8–1.5 mm PPD reduction were consistently observed, particularly in deep periodontal pockets, intrabony defects and in patients with systemic comorbidities [24,25,27,29]. These advantages align with earlier findings suggesting that HA may accelerate connective tissue repair and promote reattachment under conditions of reduced microbial activity [28,30]. Repeated HA application also appears to diminish bleeding on probing and postoperative discomfort, aligning with models of improved wound stability and reduced inflammatory burden [31,32]. Such effects are clinically relevant since persistent inflammation and tissue fragility are associated with recurrence of disease and residual pockets [33,34].

In the context of regenerative periodontal surgery, HA has also been explored alongside membranes, enamel matrix derivatives, and bone substitutes, with encouraging outcomes [35,36,37]. Although heterogeneity in application protocols and defect morphology complicates pooling of data, the overall direction of evidence supports the adjunctive regenerative role of HA [38]. However, interpretation of the reported clinical benefits of hyaluronic acid must take into account substantial heterogeneity across studies, particularly related to differences in formulation characteristics and application protocols. An important source of heterogeneity across clinical trials evaluating hyaluronic acid lies in the variability of its physicochemical properties, particularly molecular weight and degree of cross-linking. High-molecular-weight and cross-linked HA formulations exhibit prolonged tissue residence time, enhanced viscoelastic behavior, and greater resistance to enzymatic degradation, which may translate into more sustained anti-inflammatory and immunomodulatory effects at periodontal wound sites [12,16,36,38]. In contrast, low-molecular-weight HA is characterized by faster degradation kinetics and may preferentially activate different cellular signaling pathways, potentially resulting in more transient clinical effects [12,37]. These formulation-dependent differences likely contribute to the variability observed in probing depth reduction, clinical attachment gain, and bleeding on probing outcomes across studies [23,24,25,26]. The lack of standardized reporting of HA molecular characteristics and application protocols currently limits direct comparison among trials and represents a critical methodological gap that should be addressed in future randomized studies [14,15]. Importantly, the use of HA in periodontology is not solely empirical; basic science models demonstrate that HA decreases biofilm adhesion, inflammatory cytokine release, and epithelial cell damage, thereby explaining its soft-tissue stabilization effects observed clinically [12,38]. The translational link between mechanistic and clinical findings strengthens confidence in HA’s regenerative contribution [39]. Beyond hyaluronic acid, several other chemical and biologically active adjuncts have been investigated for periodontal pocket therapy and regenerative procedures. These include enamel matrix derivatives, platelet-derived preparations such as platelet-rich fibrin, recombinant growth factors, and emerging immunomodulatory biomaterials designed to influence inflammatory resolution and tissue regeneration [2,3,14,15]. While some of these approaches have demonstrated regenerative potential, their clinical application is often limited by higher biological complexity, cost, or technique sensitivity. In contrast, hyaluronic acid has gained attention due to its favorable safety profile, ease of application, and consistent anti-inflammatory and wound-stabilizing effects. The present review therefore focused on HA and β-tricalcium phosphate as representative and widely accessible adjunctive biomaterials, while acknowledging that alternative chemical strategies continue to evolve within the broader regenerative landscape.

In parallel, the role of β-TCP in alveolar ridge preservation, sinus augmentation and lateral ridge reconstruction is well established. β-TCP provides a porous osteoconductive scaffold that is gradually resorbed and replaced by vital bone, enabling dimensional maintenance and subsequent implant placement [30,31]. Numerous studies show horizontal bone gains of ~2.5–4 mm and vertical gains of ~5–7 mm in sinus elevation procedures, with implant survival exceeding 95% in long-term evaluations [30,31,32,33]. Histologic analyses frequently demonstrate vital bone proportions of ~35–40% at re-entry, confirming successful integration of β-TCP with host tissue [34,35,36]. These findings align with previous observations that β-TCP morphology, porosity and ionic dissolution kinetics facilitate bone remodeling rather than simply occupying space [37,38,39]. Beyond its well-documented osteoconductive properties, the clinical performance of β-tricalcium phosphate is also influenced by mechanical and defect-related factors that extend beyond material composition alone. From a clinical perspective, the regenerative performance of β-tricalcium phosphate must be interpreted in relation to defect morphology and mechanical stability. Although β-TCP provides an effective osteoconductive scaffold and undergoes controlled resorption coupled with new bone formation, its volume maintenance in large or non-contained defects remains dependent on adjunctive space-maintaining strategies [30,31,32]. Clinical evidence supports the use of barrier membranes, tenting screws, or combined stabilization techniques to prevent graft collapse during the resorption phase and to preserve the regenerative space necessary for predictable bone formation [33,34,35]. Failure to ensure adequate space maintenance may result in partial volume loss despite favorable biological remodeling [30,34]. Therefore, successful application of β-TCP in advanced alveolar defects should be integrated within defect-specific surgical protocols that account for both biological and mechanical requirements [31,35].

Importantly, evidence from orthopedic and craniofacial surgery demonstrates similar remodeling profiles of β-TCP in load-bearing contexts, thereby validating its translational relevance and not restricting its use to dental settings alone [40,41,42]. Controlled β-TCP degradation and osteoclast–osteoblast coupling, observed in orthopedic literature, reinforce its suitability for alveolar regeneration where long-term stability and implant osseointegration are required [43]. The parallelism between dental and orthopedic outcomes suggests that material behavior is not site-restricted but instead dependent on mechanical and metabolic conditions, supporting confidence in β-TCP use in oral environments.

When interpreting clinical outcomes, it is crucial to acknowledge the complementary nature of HA and β-TCP. HA primarily improves soft-tissue resolution and inflammatory modulation, while β-TCP promotes hard-tissue regeneration and scaffold-guided osteoconduction. Clinical scenarios frequently demand both—deep periodontal defects involve chronic inflammation alongside bone loss, whereas post-extraction sockets require soft-tissue sealing for uncontaminated bone formation. Consequently, combining these materials may be biologically synergistic, although controlled trials assessing combined application remain scarce. Nonetheless, the convergence of soft-tissue biology, scaffold science, and clinical performance suggests that such strategies warrant further investigation in future randomized trials.

Despite promising evidence, methodological limitations should be recognized. In addition, no formal grading of the certainty of evidence (e.g., GRADE approach) was performed, which limits the ability to draw hierarchical conclusions regarding strength of evidence across outcomes. Variability in follow-ups, HA formulations, β-TCP granule sizes, defect morphologies, and outcome reporting standards hinders quantitative synthesis. Many clinical studies lack long-term data beyond the implant placement stage, while in vitro models cannot fully replicate the complex inflammatory and biomechanical environment of healing defects. Furthermore, biological responses to HA and β-TCP may differ depending on systemic conditions, smoking status, and microbiome composition. These limitations underline the need for standardized protocols, harmonized reporting of clinical endpoints and integration of mechanistic biomarkers to better correlate material behavior with clinical results.

Despite these constraints, the totality of evidence supports the utility of HA and β-TCP across diverse regenerative scenarios. Their complementary biological effects—soft-tissue modulation and scaffold-mediated osteoconduction—offer a multifaceted approach suited to the demands of periodontal regeneration and ridge preservation. Future studies exploring co-delivery systems, controlled release matrices and sequential application may provide additional gains in stability and regenerative predictability. Ultimately, the integration of HA and β-TCP within personalized regenerative frameworks could improve clinical outcomes, reduce complication rates and enhance predictability in implant rehabilitation.

The interpretation of clinical trends must be moderated by methodological limitations, including unclear allocation procedures and restricted blinding in several trials, which may inflate observed effect sizes. Furthermore, heterogeneity in outcome metrics and follow-up periods precludes direct quantitative synthesis. These quality considerations constrain the strength of inference and highlight the need for standardized reporting in future investigations.

## 5. Conclusions

Adjunctive hyaluronic acid (HA) demonstrates consistent additional improvements when combined with conventional periodontal therapy and indicates potential clinical benefit, particularly in deeper periodontal sites and in medically compromised patients. These observed trends align with translational in vitro findings showing that HA modulates inflammation, supports fibroblast and epithelial activity, and stabilizes early soft-tissue healing; however, the magnitude of benefit remains difficult to quantify due to heterogeneous study designs, outcome measurements and follow-up durations. Accordingly, HA should currently be regarded as a promising adjunctive modality rather than a definitive regenerative intervention.

β-Tricalcium phosphate (β-TCP) provides predictable scaffold-mediated bone regeneration, supporting alveolar ridge preservation, sinus augmentation and subsequent implant placement. Clinical dimensional stability and histologic evidence of scaffold-guided bone formation are biologically reinforced by mechanistic data demonstrating controlled osteoclast-mediated resorption and osteoblast differentiation. While overall findings support β-TCP as a reliable osteoconductive biomaterial, variability in graft configuration, membrane usage, and adjunctive biologics underscores the need for standardized reporting to clarify clinical effect size and timing of optimal scaffold remodeling.

Taken together, HA and β-TCP exhibit complementary regenerative roles across soft- and hard-tissue compartments: HA facilitates early wound stability through inflammatory modulation and tissue reorganization, while β-TCP maintains structural space to enable scaffold-guided new bone formation and implant feasibility. The sequential interplay between soft-tissue resolution and scaffold-mediated osteogenesis provides a biologically rational foundation for future protocols combining or staging these materials.

Nevertheless, interpretation must be tempered by moderate risk of bias across included studies, particularly regarding limited blinding, allocation concealment and outcome heterogeneity, which may influence the strength of inference. Future randomized controlled trials with standardized endpoints, longer follow-ups, and biomarker-based mechanistic readouts are necessary to determine whether integrated or sequential use of HA and β-TCP can enhance regenerative predictability and improve long-term periodontal and implant stability.

## Figures and Tables

**Table 1:** Characteristics of included clinical studies—HA in periodontal pocket therapy.

Study (Year)	Study Design	Intervention (Test)	Control/Comparison	Key Outcomes (Justified)
Olszewska-Czyz et al. [24]	Randomized, controlled clinical trial	Adjunctive HA gel + SRP	SRP alone	This RCT evaluated HA as an adjunct in moderate periodontitis and reported improved periodontal healing parameters (e.g., PPD and CAL) compared to control.
Aydinyurt et al. [25]	Prospective clinical study	HA gel adjunct to SRP	SRP alone	Early effects of HA adjuvant therapy demonstrated favorable changes in clinical parameters (e.g., PPD and PISA) and biochemical markers, suggesting adjunctive benefit.
Ezer & Gunpinar et al. [26]	Randomized clinical trial	0.8% HA gel adjunct to MINST (intrabony defects)	MINST alone	Reported no additional benefit for main clinical/radiographic outcomes versus control but relevant as a controlled human trial highlighting formulation/protocol-dependence.
Olszewska-Czyz et al. [27]	3-Month randomized clinical trial	HA gel adjunct	Placebo or standard care	Local adjunctive HA in diabetic patients with periodontitis improved CAL gain and BOP reduction, indicating efficacy in compromised populations.
Al-Abbadi et al. [28]	Randomized controlled trial	Topical HA gel + professional mechanical plaque removal	PMPR alone	The trial in diabetic periodontitis patients suggested benefits of HA in improving clinical and radiographic outcomes, though full publication awaits peer-review.
Rodríguez-A et al. [29]	18-Month RCT	HA with minimally invasive techniques	Standard therapy	Demonstrated enhanced PPD reduction, CAL gain, and radiographic bone fill compared to control, supporting HA’s regenerative potential.

Notes: These clinical studies evaluate the adjunctive effect of HA in non-surgical periodontal therapy, focusing on outcomes like probing depth, clinical attachment level, inflammation markers, and radiographic changes.

**Table 2:** Characteristics of included clinical studies—β-TCP in alveolar bone augmentation.

Study (Year)	Augmentation & Design	Intervention (Test)	Comparison	Key Outcomes (Justified)
Velasco-Ortega et al. [30]	Long-term clinical study of maxillary sinus augmentation	β-TCP graft in lateral sinus approach with implants	No comparator/single cohort	Over a long-term follow-up (up to 10 years), implants placed with β-TCP showed high survival and stable bone regeneration post-sinus augmentation.
Han et al. [31]	Multicenter RCT	rhBMP-2 + β-TCP in ridge preservation	β-TCP alone	rhBMP-2/β-TCP provided enhanced alveolar ridge preservation effects compared with β-TCP alone, with similar safety profiles.
Lamas et al. [32]	Clinical evaluation of sinus lift	β-TCP graft with simultaneous implant placement	-	β-TCP achieved substantial vertical bone gain in maxillary sinus lift procedures, with implant success rates similar to grafted sites.
Joshi et al. [33]	Prospective, controlled clinical study of socket preservation	β-TCP in extraction sockets	Autogenous graft/ungrafted controls	Compared β-TCP alloplast to other ridge preservation options; β-TCP maintained alveolar dimensions with acceptable bone density.
Al Shawaheen et al. [34]	Clinical ridge splitting study	Particulate β-TCP graft	-	β-TCP enhanced bone width recovery in mandibular ridge splitting, showing feasible clinical use for horizontal augmentation.
Saito et al. [35]	Multicenter randomized controlled trial (alveolar ridge preservation)	PLGA-coated β-TCP (moldable alloplastic graft)	FDBA + rapidly absorbable collagen dressing	Both approaches maintained ridge dimensions compatible with implant site development; histomorphometric outcomes differed in the proportion of mineralized tissue, without compromising clinical feasibility.

Notes: β-TCP is commonly used in sinus lift, ridge preservation, and augmentation procedures. The trials listed reflect real clinical usage of β-TCP grafts with measurable bone regeneration outcomes.

**Table 3:** Overview of included in vitro studies on HA and β-TCP.

Study (Year)	Cell/Model Type	Material Evaluated	Experimental Setting	Key Findings
Fujioka-Kobayashi et al. [36]	Human periodontal ligament cells (PDL)	Hyaluronic acid (HA)	HA (cross-linked and non-cross-linked) effects on cell viability, proliferation, differentiation	Both HA formulations maintained high PDL viability and increased proliferation; early osteogenic differentiation markers were increased, though late osteogenic markers varied, indicating HA’s supportive role in early periodontal regeneration processes.
X Zhu et al. [37]	Periodontal biofilm + MONO-MAC-6 & PDL fibroblasts	Four different HA types (HHA, LHA, OHA, and CHA)	Interaction of HA with multispecies periodontal biofilm and immune/PDL cells	High- and cross-linked HA reduced biofilm bacterial counts and modulated inflammatory cytokine expression in immune and PDL cells, suggesting anti-biofilm and anti-inflammatory potential relevant to periodontal microenvironments.
Barbieri et al. [38]	Gingival epithelial cells, osteoblasts, PDL fibroblasts	High-molecular-weight HA	HA impacts on viability, proliferation, gene expression in co-culture	HA promoted overall cell viability and influenced osteogenic gene expression (e.g., ALP and CD44), highlighting HA’s biocompatibility and potential regenerative effects on multiple periodontal cell types.
Hakki et al. [39]	Oral tissue cells (PDL and gingival fibroblasts)	HA	HA impact on migration, viability, and cellular responses	In vitro studies support HA’s role in enhancing PDL cell proliferation, migration and viability, indicating suitability for periodontal wound healing and regeneration protocols.
Beuvelot et al. [40]	Osteoblast-like (SaOS-2) and macrophages	β-TCP particles	β-TCP particle contact with cells in long-term culture	SaOS-2 proliferated actively toward β-TCP particles and macrophages interacted with surfaces, demonstrating β-TCP’s biocompatibility and potential to support osteogenesis and cell colonization in bone defect environments.
Zheng et al. [41]	MC3T3-E1 (osteoblast) and TRAP osteoclasts	Surface-charged β-TCP	β-TCP effects on osteoblast and osteoclast differentiation and resorption	Charged β-TCP surfaces influenced TRAP activity and osteoclast morphology, illustrating surface chemistry effects on osteoclastogenesis and β-TCP resorption relevant to remodeling in bone regeneration.
Lu et al. [42]	MG-63 osteoblast-like cells	β-TCP + bioactive glass composite	Composite scaffolds with β-TCP stimulated higher osteoblast proliferation than control	Composite β-TCP/BG scaffold stimulated osteoblast proliferation more than pure β-TCP, suggesting that β-TCP composites may enhance osteogenic support in vitro.
Zhu et al. [43]	Periodontal biofilm model + periodontal ligament fibroblasts	Hyaluronic acid (cross-linked HA) ± human serum	Multi-species biofilm challenge with inflammatory readouts (e.g., IL-8) and cellular response assessment	Cross-linked HA (±serum) reduced biofilm activity and modulated inflammatory responses in periodontal ligament fibroblasts, supporting anti-biofilm and immunomodulatory relevance.

Notes: HA = hyaluronic acid; β-TCP = beta-tricalcium phosphate; PDL = periodontal ligament cells; MG-63 = human osteoblast-like cell line; MC3T3-E1 = mouse calvaria-derived pre-osteoblasts; TRAP = tartrate-resistant acid phosphatase (osteoclast marker); BG = bioactive glass.

## Data Availability

The original contributions presented in this study are included in the article and Supplementary Material. Further inquiries can be directed to the corresponding author.

## Supplementary Materials

The following supporting information can be downloaded at: https://www.mdpi.com/article/10.3390/dj14020097/s1. File S1: PRISMA 2020 Checklist (completed). File S2: Full Electronic Search Strategies (all databases). File S3: List of Excluded Full-Text Studies with Reasons. File S4: PRISMA 2020 Flow Diagram (graphical version). File S5: Risk of Bias and Quality Assessment Details.
