# Supplementary material for: Hyaluronic Acid and β-Tricalcium Phosphate in Periodontal Pocket Therapy and Alveolar Bone Augmentation: A Systematic Review
Source: Dent J (Basel). 2026 Feb 10;14(2):97. doi: 10.3390/dj14020097 (PMC12939553; doi:10.3390/dj14020097)
Supplement: Supplementary file 1 [file dentistry-14-00097-s001.zip › Supplementary_File_S2_Search_Strategies.pdf]

Supplementary File S2

Full Electronic Search Strategies

PubMed (MEDLINE)

("Hyaluronic Acid"[Mesh] OR "hyaluronic acid" OR hyaluronan) AND (periodont* OR "periodontal pocket" OR "intrabony defect") OR (beta-tricalcium phosphate OR β-tricalcium phosphate OR tricalcium phosphate) AND ("alveolar ridge augmentation" OR "bone augmentation" OR "socket preservation" OR "sinus lift") Filters: English language, 2015–2025

Scopus

TITLE-ABS-KEY("hyaluronic acid" OR hyaluronan) AND (periodontal OR intrabony) OR TITLE-ABS-KEY("beta-tricalcium phosphate" OR β-TCP) AND ("alveolar ridge augmentation" OR "bone regeneration")

Web of Science

TS=(hyaluronic acid OR hyaluronan) AND TS=(periodontal) OR TS=(beta-tricalcium phosphate OR β-TCP) AND TS=(alveolar bone)

Embase

('hyaluronic acid'/exp OR hyaluronan) AND (periodontal disease) OR ('beta tricalcium phosphate'/exp) AND (alveolar bone regeneration)
