# Supplementary material for: Hyaluronic Acid and β-Tricalcium Phosphate in Periodontal Pocket Therapy and Alveolar Bone Augmentation: A Systematic Review
Source: Dent J (Basel). 2026 Feb 10;14(2):97. doi: 10.3390/dj14020097 (PMC12939553; doi:10.3390/dj14020097)
Supplement: Supplementary file 1 [file dentistry-14-00097-s001.zip › Supplementary_File_S3_Excluded_Studies.pdf]

Supplementary File S3 (Revised)

List of Excluded Full-Text Studies with Reasons

Author (Year)	Reason for Exclusion
Casale et al. (2016)	Review article (not primary research)
Malcangi et al. (2023)	Systematic review (not eligible study type)
Chavda & Levin (2018)	Review article (no original clinical or in vitro data)
Suzuki et al. (2015)	Animal study (non-human subjects)
Simion & Fontana (2004)	Published outside predefined time frame (before 2015)
Singh et al. (2019)	Outcomes not relevant to predefined review outcomes
Mazzuffi et al. (2015)	Preclinical animal model
