# Supplementary material for: Hyaluronic Acid and β-Tricalcium Phosphate in Periodontal Pocket Therapy and Alveolar Bone Augmentation: A Systematic Review
Source: Dent J (Basel). 2026 Feb 10;14(2):97. doi: 10.3390/dj14020097 (PMC12939553; doi:10.3390/dj14020097)
Supplement: Supplementary file 1 [file dentistry-14-00097-s001.zip › Supplementary_File_S4_PRISMA_Flow_Diagram_Graphical.pdf]

Supplementary File S4

PRISMA 2020 Flow Diagram

**Identification**	
Records identified through database searching (n = 312)	PubMed (98)<br>Scopus (87)<br>Web of Science (64)<br>Embase (63)
Records after duplicates removed (n = 241)	
Screening	
Records screened (n = 241)	Records excluded (n = 179)
Eligibility	
Full-text articles assessed (n = 62)	Full-text articles excluded (n = 42)<br>- Wrong study design: 18<br>- Irrelevant outcomes: 12<br>- Insufficient data: 7<br>- Non-English: 5
Included	
Studies included in systematic review (n = 20)	Clinical studies: 12<br>In vitro studies: 8
