# Supplementary material for: Hyaluronic Acid and β-Tricalcium Phosphate in Periodontal Pocket Therapy and Alveolar Bone Augmentation: A Systematic Review
Source: Dent J (Basel). 2026 Feb 10;14(2):97. doi: 10.3390/dj14020097 (PMC12939553; doi:10.3390/dj14020097)
Supplement: Supplementary file 1 [file dentistry-14-00097-s001.zip › Supplementary_File_S5_Risk_of_Bias.pdf]

Supplementary File S5

Risk of Bias and Quality Assessment

A. Randomized Controlled Trials – Cochrane RoB 2.0

Study	Randomization	Deviations	Missing Data	Measurement	Reporting	Overall
Pilloni et al. (2021)	Low	Some concerns	Low	Low	Low	Some concerns
Mamajiwala et al. (2021)	Low	Some concerns	Low	Low	Low	Some concerns
Nguyen et al. (2021)	Some concerns	Some concerns	Low	Low	Low	Some concerns

B. Observational Studies – Newcastle–Ottawa Scale

Study	Selection	Comparability	Outcome	Total Score
Monsalve-Guil et al. (2021)	★★★	★★	★★★	8/9
Joshi et al. (2016)	★★★	★★	★★	7/9

C. In Vitro Studies – Methodological Quality Assessment

In vitro studies were assessed based on clarity of objectives, use of appropriate controls, replicability of experiments, and clinical relevance of the experimental model. Overall methodological quality ranged from moderate to high. Common limitations included short observation periods and simplified experimental conditions.
